# Factors Influencing Postoperative Overactive Bladder after Adjustable Trans-Obturator Male System Implantation for Male Stress Incontinence following Prostatectomy

**DOI:** 10.3390/jcm12247505

**Published:** 2023-12-05

**Authors:** Miguel Virseda-Chamorro, Carlos Téllez, Jesus Salinas-Casado, Juliusz Szczesniewski, Sonia Ruiz-Grana, Ignacio Arance, Javier C. Angulo

**Affiliations:** 1 Urology Department, Hospital Nacional de Parapléjicos, 45071 Toledo, Spain; mvirseda@sescam.jccm.es; 2 Urology Department, Hospital Universitario de Getafe, 28905 Getafe, Spain; carlos.tellez@salud.madrid.org (C.T.); juliuszjan.szczesniewski@salud.madrid.org (J.S.); srgrana@salud.madrid.org (S.R.-G.); ignacio.arance@salud.madrid.org (I.A.); 3 Clinical Department, Faculty of Medical Sciences, Universidad Europea, 28670 Madrid, Spain; 4 Urology Department, Hospital Clínico de San Carlos, 28040 Madrid, Spain; jesus.salinas@salud.madrid.org; 5 Department of Surgery, Faculty of Medicine, Universidad Complutense, 28040 Madrid, Spain

**Keywords:** overactive bladder, postprostatectomy urinary incontinence, detrusor overactivity, stress urinary incontinence, male sling

## Abstract

We aimed to determine the risk factors for postoperative overactive bladder (OAB) in patients treated with an adjustable trans-obturator male system (ATOMS) for stress incontinence after radical treatment of prostate cancer. A prospective study was performed on 56 patients implanted with an ATOMS for PPI. Clinical and urodynamic information was recorded before and after ATOMS implantation. We built a multivariate model to find out the clinical and urodynamic factors that independently influenced postoperative OAB and the prognostic factors that influenced the efficacy of medical treatment of OAB. We found that the clinical risk factors were the preoperative intensity of urinary incontinence (number of daily pads used and amount of urinary leakage), International Consultation on Incontinence Questionnaire (ICIQ) score, postoperative number of ATOMS adjustments, final cushion volume, and incontinence cure. The urodynamic data associated with OAB were cystometric bladder capacity, voided volume, volume at initial involuntary contraction (IC), maximum flow rate, bladder contractility index (BCI), and urethral resistance (URA). The prognostic factors for the efficacy of oral treatment of OAB were the volume at the first IC (direct relationship) and the maximum abdominal voiding pressure (inverse relationship). The multivariate model showed that the independent clinical risk factors were the daily pad count before the implantation and the ICIQ score at baseline and after treatment. The independent urodynamic data were the volume at the first IC (inverse relationship) and the URA value (direct relationship). Both predictive factors of treatment efficacy were found to be independent. Detrusor overactivity plays an important role in postoperative OAB, although other urodynamic and clinical factors such as the degree of urethral resistance and abdominal strength may influence this condition.

## 1. Introduction

Male stress urinary incontinence (SUI) after prostatectomy is one of the most devastating complications of prostate surgery. This complication greatly affects the quality of life of patients and originates after partial or total injury of the urethral sphincter mechanism [1,2]. Prosthetic surgery is recommended when pelvic floor training does not allow continence recovery, but the surgical options can be different depending on the degree of sphincteric damage and the available alternatives [3]. Total damage of the urinary sphincter is best treated with an artificial urinary sphincter (AUS), but male sling techniques have been used in the last decades for partial sphincteric lesions based on their similar efficacy and diminished complications in the long term [4,5].

One of these sling approaches, an adjustable trans-obturator male system (ATOMS), is being increasingly used to treat mild-to-moderate SUI. It is an effective and safe device, with a limited rate of postoperative complications. In addition, it does not need patient manipulation [6,7]. An increased body of clinical evidence has been developed for this continence system in the last decade. However, some questions remain unsolved. One of these is that with an unknown frequency, some patients implanted with an ATOMS develop de novo overactive bladder (OAB), and this topic may have been under-considered in the recent medical literature [8]. In other patients, OAB is present at baseline, together with SUI, and remains to some extent after the implantation. We also do not know if this coexistence of stress and urge incontinence has prognostic implications for prosthetic surgery in these patients, as OAB may remain or disappear after surgery.

According to the International Continence Society (ICS), OAB symptoms include urgency, increased urinary frequency, and sometimes urge incontinence [9]. This condition can significantly impact patients’ quality of life and may be associated with other comorbidities [10]. The real prevalence of OAB symptoms in patients suffering from SUI after prostatectomy is unknown. There is extensive controversy about the risk factors for postoperative OAB after surgical repair of male SUI. Urinary urgency may resolve after AUS implantation, provided patients were not previously irradiated [11]. On the other hand, some authors could not find any relationship between preoperative OAB and the global outcome [12], whereas other authors found that improvement in urgency after prosthetic surgery was associated with curing SUI [13].

Therefore, we intended to evaluate which are the clinical and urodynamic factors that are associated with the presence of OAB symptoms in a series of patients with post-prostatectomy SUI intervened with an ATOMS. For that purpose, we compared the populations with and without postoperative OAB symptoms to elucidate the clinical and urodynamic factors involved. We also intended to evaluate the prognostic factors that influence the efficacy of medical treatment for OAB symptoms in these patients. Lastly, we focused on the urodynamic parameters in baseline evaluations that appear associated with postoperative OAB after ATOMS implantation [14].

## 2. Materials and Methods

We performed a prospective longitudinal study between October 2020 and March 2021. We evaluated a cohort of patients with primary ATOMS implantations for SUI after refractory prostate cancer surgery to conservative rehabilitation techniques. The inclusion criteria included a urodynamic study performed before the ATOMS implantation and a follow-up after ATOMS surgery of longer than 12 months. All patients gave signed informed consent. The exclusion criteria included a need to undergo endourological surgery for stone surgery or a bladder tumor that obliged modifying the ATOMS system filling decided at the time of the final ATOMS adjustment and the impossibility of performing postoperative urodynamic evaluation for technical reasons. The sample size was calculated according to the study by Toia et al. [15] performed with a retrobulbar sling. According to that study, we needed a minimum sample size of 54 patients to find the baseline OAB in 27% of continent patients and 55% of incontinent ones after retro-urethral sling implantation, with an alpha error of 5% and a statistical power of 80%.

The medical record of each patient was reviewed. The severity of baseline incontinence was registered for each patient, according to a 24 h pad count and 24-pad test, and also the preoperative International Consultation on Incontinence Questionnaire (ICIQ) before the ATOMS implantation. We registered the incontinence severity and the ICIQ questionnaire again postoperatively after the ATOMS adjustment. Also, variables such as the number of ATOMS fillings needed for adjustment and the final cushion volume reached were evaluated. Late postoperative complications were defined as any situation that affected the continence status or any event related to the surgical implant, graded in accordance with the common terminology criteria for adverse events (CTCAE v5.0), and also the surgical and medical procedures performed on the patient after the implantation [16]. The presence of OAB symptoms at the last follow-up was also recorded.

Treatment success in terms of continence after device adjustment was defined as no need for pads or use of one security pad/day with less than 10 mL of urine loss. Patients in which postoperative symptoms of OAB were detected initiated oral treatment with the antimuscarinic medication solifenacin (5 mg once daily) and reviewed three months later to evaluate the effect of this treatment and whether it should be continued.

The urodynamic study was performed with the same standards and included filling cystometry in the filling phase and pressure flow study in the voiding phase. The polygraph used was Uro 2000 (Medical Measure System, Enschede, The Netherlands). This study was performed and evaluated according to the specifications of the International Continence Society (ICS) [17] and to the protocols of Good Urodynamic Practices (GUP) [18]. Each patient was placed in a standing position, and bladder filling through an 8 Fr two-way transurethral catheter with saline solution at room temperature at a rate of 50 mL/s was performed. The abdominal pressure was recorded using a transrectal balloon catheter, and the abdominal and bladder pressures were measured with reference to the atmospheric pressure. Voiding was performed in a standing position after the patient reported a strong desire to void or registered a terminal involuntary detrusor contraction. Bladder outlet obstruction (BOO) was registered according to Bladder Outlet Obstruction Index (BOOI) and also to the Urethral Resistance Factor (URA). Also, detrusor contractility was calculated according to the Bladder Contractility Index (BCI). Following these parameters, bladder outlet obstruction was diagnosed when the URA value was equal to or greater than 29 cm H_2_O, and detrusor underactivity was diagnosed whenever the BCI value was lower than 100 cm H_2_O.

All the ATOMS devices implanted were of the pre-attached silicone-covered scrotal port design, following the original surgical description [6,19]. The procedure was performed under spinal anesthesia with the patient in a lithotomy position, after the insertion of a 14 Fr Foley urethral catheter for bladder drainage. The trans-obturator passage of the device was performed using helical tunnelers. After knotting the mesh arms to the silicone cushion, the device established a four-point sub-urethral fixation that compressed the bulbo-membranous urethra. The cushion filling was performed in the operative room up to atmospheric pressure and postoperative additional filling of the cushion reservoir was performed via direct percutaneous injection of physiological sodium chloride into the scrotal port. Postoperative adjustment was performed 1 month after the implantation and was repeated periodically if needed until either dryness was achieved or the maximum filling capacity of the system was reached [19]. The ATOMS filling after adjustment was registered in ml. Data were included in an institutional-review-board-approved database regarding not only clinical data but also the urodynamic findings. Tabulation of urodynamic data was intentionally independent from that of baseline clinical data, and also independent from the register of the postoperative outcomes.

Statistical analysis was performed using the IBM^®^ SPSS^®^ Statistics 22.0 software. The tests of statistical significance used were a mean comparison test for dependent groups (Student’s *t*-test) for parametric variables and Wilcoxon’s signed-rank test for nonparametric variables. The parametric distribution of variables was tested using the Kolmogorov–Smirnoff test. Stepwise multivariate logistic analysis was performed to evaluate the variables that independently influenced the presence of postoperative OAB symptoms. The quantitative variables are expressed as means ± standard deviation and the qualitative variables by absolute number and percentage.

## 3. Results

A total of 84 patients were screened for this study. Three patients were excluded because they died of another disease during follow-up. Nineteen patients did not give consent to undergo a postoperative urodynamic study. Another three patients had urinary tract surgery performed after the ATOMS implantation (transurethral resection of a bladder tumor in two patients and ureteroscopy in one). Additionally, two patients had an irregular urethra in which the urodynamic catheter could not be inserted, and another patient was not able to urinate because of perineal contraction. Consequently, the final sample size of patients included in the study was 56 cases with a mean age of 70 ± 6 years. Four of these patients (7%) had also received radiotherapy as part of their prostate cancer treatment. The mean number of ATOMS adjustments performed was 1.4 ± 1.6. Continence was achieved in 36 patients (64%) and postoperative complications developed in 6 (11%).

The number of patients who had preoperative OAB before ATOMS implantation was 17 (30%). Of these, nine patients (53%) maintained OAB symptoms postoperatively. Globally, OAB symptoms after ATOMS implantation were observed in 24 patients (43%), and de novo urgency appeared in 15 (27%). Eighteen patients with OAB symptoms (i.e., 32% of the study population and 75% of those with postoperative OAB symptoms) were treated with oral solifenacin during follow-up.

Table 1 compares the clinical and urodynamic data pre- and postoperatively in the series evaluated. We found the following statistically significant differences. According to the continence outcome data, the number of daily pads (pad count) and the incontinence amount (pad test) were less postoperatively. Regarding the urodynamic data, the frequency of bladder outlet obstruction, the maximum detrusor pressure (Pmax), the bladder outlet obstruction index (BOOI), and the URA were higher postoperatively. We could not confirm a significant difference in the proportion of patients with symptoms of OAB and/or detrusor overactivity (DO) before and after the implantation. Similarly, the proportion of patients with detrusor underactivity or an acontractile detrusor did not significantly worsen after the implantation. Notably, a significant reduction in cystometric capacity was evidenced, without changes in voided volume, postvoid residual, or maximum urine flow (Qmax).

Table 2 shows the distribution of OAB symptoms in the total series evaluated before and after ATOMS implantation.

Table 3 shows the relationship between preoperative clinical and urodynamic variables and postoperative overactive bladder syndrome. A significant direct relationship was observed between postoperative OAB and the clinical variables related to the severity of preoperative incontinence: ICIQ score, pad count, and pad test.

The relationships between postoperative clinical and urodynamic variables and postoperative overactive bladder are shown in Table 4. A significant direct relationship was observed between clinical variables and postoperative OAB, number of ATOMS adjustments, final cushion volume, ICIQ score, and postoperative complications and the urodynamic variables and postoperative OAB, cystometric capacity, detrusor overactivity, volume at first involuntary contraction (IC), maximum flow rate (Qmax), the urethral resistance parameter URA, and the bladder contractility index (BCI).

Regarding the treatment of postoperative OAB symptoms, the variables that significantly influenced the outcome were the volume at first involuntary contraction (IC) (134 ± 55.8 mL for patients with a positive outcome vs. 74 ± 55.3 mL for those with a negative outcome; *p* = 0.046) and the maximum abdominal voiding pressure (34 ± 26.9 cm H_2_O for patients with a positive outcome vs. 69 ± 11.0 cm H_2_0 for those with a negative outcome; *p* = 0.027). Logistic regression showed that both parameters are independent variables.

When the preoperative and postoperative clinical variables were evaluated using logistic regression, daily preoperative pad count and both preoperative and postoperative ICIQ scores were independent variables to predict the presence of postoperative OAB symptoms. The regression model of the postoperative urodynamic variables is shown in Table 5. The only independent variables were the volume at first IC, an inversely related factor, and the postoperative URA, which was directly related.

## 4. Discussion

OAB is common in men undergoing prostatic surgery because bladder outlet obstruction produces changes in the bladder wall, both in the detrusor muscle and in the urothelium, thus resulting in OAB symptoms. The prevalence of OAB in men with benign prostatic enlargement is up to 60% before prostatic resection (TURP) and may persist in 30% to 50% after TURP [20]. On the other hand, the appearance of de novo OAB after prostate surgery is not infrequent. Thirty-four percent of patients who underwent radical prostatectomy (RP) without stress urinary incontinence reported de novo OAB, which persisted in twenty-six percent of them up to 24 months post-RP [21]. This may pose a serious problem regarding the success of surgical devices implanted to correct SUI, as mixed incontinence or pure urge incontinence may limit the results achieved by the implant, and a different form of urinary incontinence may be noticed after surgery.

Consequently, postoperative OAB after surgical repair of male SUI is an issue of paramount importance regarding the quality of life, satisfaction, and global outcomes of prostate cancer survivors. In fact, OAB symptoms persist in many patients with mixed urinary incontinence after placement of an AUS [22], and most are dissatisfied even after successful repair of stress incontinence [23]. Other series have also found a relationship between continence and lower urinary tract symptoms (LUTSs) after PPI treatment. For instance, Yiou et al. [13] reported that patients who underwent male TOMS sling implantation for PPI and improved their incontinence had fewer LUTSs and better quality of life scores. Utomo et al. [24] found that prior heavier incontinence was associated with a negative clinical outcome after ProACT implantation.

As far as we know this has never been evaluated in patients with SUI treated with ATOMS. There are three main findings derived from our study. First of all, the clinical risk factors independently associated with postoperative OAB are the preoperative incontinence intensity and preoperative and postoperative ICIQ score. Secondly, urodynamic findings independently associated with postoperative OAB are the degree of detrusor overactivity and urethral resistance. Thirdly, patients who responded to medical treatment were those with less detrusor overactivity and less abdominal strength.

Contrary to our findings other authors have found that prior radiation is a risk factor for postoperative OAB after artificial sphincter implantation [23]. This difference can be attributed to the higher frequency of preoperative OAB in patients undergoing radiotherapy of this series compared with the non-irradiated group. Although it is possible that preoperative radiotherapy may influence de novo postoperative OAB [25].

Age has been associated with postoperative OAB in patients who underwent radical prostatectomy [26]. However, we did not find any relationship between age and postoperative OAB. Other studies have not found any relationship between both parameters after PPI surgery either [23]. From the urodynamic point of view, OAB syndrome is a clinical entity that is related to detrusor overactivity (DO). Although it is well accepted that not all patients who report OAB symptoms have DO, we know that most patients with OAB do have DO [27].

In our series, only postoperative DO was related to postoperative OAB (but not preoperative DO). Other studies have also found that preoperative DO does not influence postoperative outcomes in patients undergoing male bone anchoring to treat PPI [11]. However, Son and Kim [22] found that patients with a low cystometric capacity and preoperative DO had more severe postoperative LUTS, including urgency, after AUS implantation. Furthermore, their multivariate analysis showed that DO was more associated with post-AUS urgency than with any other preoperative parameter. Ko et al. [26] also reported that a low preoperative cystometric capacity was a predictive factor of de novo OAB, and Lai et al. [21] showed that patients with a low preoperative bladder capacity were more likely to have OAB after AUS placement but not to develop de novo OAB. Our patients with postoperative OAB showed a higher percentage of preoperative DO, although this difference did not reach statistical significance. It is possible that a larger sample would have reached a statistically significant difference. In patients implanted with an AUS, the postoperative relationship between DO and OAB has already been confirmed [28], but the number of patients included in that series was more than 500. This strong relationship between both parameters implies that it would be very important to study in depth the factors that influence the presence of DO in patients after PPI repair. Contrary to the proven relationship between OAB and DO, SUI did not have an influence on postoperative OAB. None of the previous studies [11,21,22,25] reported any relationship between abdominal leak point pressure or the persistence of stress incontinence and postoperative OAB.

As we have said above, DO is the main cause of OAB, and most of the patients who suffer symptoms of OAB have DO in a urodynamic study. In fact, in the present study, only 12% of the patients with postoperative OAB did not have DO. Although we found that urodynamic parameters other than DO were related to postoperative OAB, multivariate analysis showed that there was only one independent factor related to postoperative OAB. This factor was the degree of urethral resistance. Increased urethral resistance leads to bladder outlet obstruction (BOO). It can be hypothesized that BOO may induce OAB through cholinergic denervation of the detrusor and subsequent hypersensitivity to acetylcholine and other neurotransmitters [29]. On the other hand, other studies consider that patients with OAB symptoms and the absence of DO have an altered response to sympathetic tests [30]. In this regard, bladder sensors are responsible for urgency. These data support the observation that patients with OAB but without DO have a defect in afferent stimuli due to increased urethral resistance [31].

In as much, DO also has a role in the response to oral therapy in patients with postoperative OAB symptoms. Patients with a positive response probably have a lower degree of DO, as confirmed by a greater volume at first involuntary contraction, than patients who do not. Another independent risk factor that could have an influence on the response is the intensity of abdominal contraction during voiding. In fact, abdominal strength is related to dysfunctional voiding, and patients who respond to treatment have fewer abdominal contractions than those who do not respond. It must also be taken into account that patients who have been previously treated with a radical prostatectomy have denervation and reinnervation phenomena in their pelvic floor muscles, also somehow responsible for dysfunctional voiding [32]. This can also affect their response to postoperative OAB treatment.

The main limitation of the current study is the high percentage of patients among the screened population who for several reasons did not undergo a second urodynamic study, which could lead to a selection bias. On the contrary, the strength of our study is the possibility of comparing preoperative and postoperative urodynamic data, which allows us to obtain objective data on lower urinary tract dysfunctions associated with postoperative OAB in patients submitted to surgical treatment of male incontinence after radical prostatectomy.

## 5. Conclusions

In conclusion, we can state that DO plays an important role in postoperative OAB in patients undergoing ATOMS implantation for PPI treatment, although other urodynamic and clinical factors may influence this condition, such as the degree of preoperative incontinence and the degree of urethral resistance and abdominal strength.

## Figures and Tables

**Table 1:** Comparison between preoperative and postoperative variables in the total series (*n* = 56).

	Preoperative	Postoperative	Significance
Pad number per day *	4.7 ± 1.95	0.5 ± 0.91	0.000 ‡
Incontinence amount (mL/day) †	620 ± 377.2	40 ± 84.4	0.000 ‡
OAB *	17 (30%)	24 (43%)	0.237
Qmax in uroflowmetry (mL/s) †	13 ± 8.7	12 ± 12.1	0.887
Voiding volume in uroflowmetry (mL) †	126 ±122.4	185 ± 192.6	0.464
Postvoiding residual in uroflowmetry (mL) †	38 ± 96.6	24 ± 52.0	0.729
Cystometric capacity (mL) †	276 ± 107.9	200 ± 102.0	0.000 ‡
Cystometric capacity (mL) †	276 ± 107.9	200 ± 102.0	0.000 ‡
DO *	30 (54%)	40 (71%)	0.127
Abdominal leak point pressure (cm H_2_O) †	75 ± 29.5	152 ± 73.9	0.009 ‡
Pmax (cm H_2_O) †	29 ± 26.8	40 ± 28.2	0.012 ‡
PQmax (cm H_2_O) †	21 ± 20.5	28 ±21.6	0.095
BOOI (cm H_2_O) †	−7.1 ± 27.73	8.6 ± 29.70	0.001 ‡
URA (cm H_2_O) †	11.9 ± 1305	17.7 ± 14.85	0.004 ‡
BCI (cm H_2_O) †	92.8 ± 46.98	74.7 ± 41.97	0.020 ‡
BOO *	5 (9%)	10 (18%)	0.037 ‡
Detrusor underactivity *	33 (59%)	45 (80%)	0.17
Acontractile detrusor *	15 (27%)	9 (16%)	0.229

* Number (percentage); † mean ± standard deviation; ‡ significant; OAB: overactive bladder; Qmax: maximum flow rate; DO: detrusor overactivity; Pmax: maximum voiding detrusor pressure; PQmax: detrusor pressure at Qmax; BOOI: bladder outlet obstruction index; URA: urethral resistance; BCI: bladder contractility index; BOO: bladder outlet obstruction.

**Table 2:** Distribution of OAB symptoms before and after ATOMS implantation (*n* = 56).

	Postoperative OAB (*n* = 24)	No Postoperative OAB (*n* = 32)	*n*
Baseline OAB symptoms present †	9	8	17
No baseline OAB symptoms †	15 ‡	24	39
Total series			56

† Before ATOMS implantation; ‡ patients with de novo OAB.

**Table 3:** Relationship between preoperative clinical and urodynamic variables and presence of postoperative detrusor overactivity.

	Postoperative OAB (*n* = 24)	No Postoperative OAB (*n* = 32)	Significance
Radiotherapy history *	2 (8%)	2 (6%)	0.578
Age (years) †	69 ± 6.5	71 ± 5.7	0.42
Pad number per day †	6 ± 1.9	4 ± 1.7	0.004 ‡
ICIQ score †	16 ± 3.2	14 ± 2.5	0.007 ‡
OAB *	9 (37%)	8 (25%)	0.237
Incontinence amount (mL/day) †	786 ± 456.6	284 ± 107.3	0.016 ‡
Cystometric capacity (mL) †	265 ± 110.0	265 ± 130.3	0.516
Bladder compliance (mL/cm H_2_O) †	123 ± 132.9	98 ± 105.6	0.436
Detrusor overactivity *	16 (67%)	14 (44%)	0.076
Volume at first IC (mL) †	222 ± 71.2	219 ± 77.3	0.907
Maximum IC pressure (cm H_2_O) †	51 ± 26.3	35 ± 23.7	0.103
Abdominal leak point pressure (cm H_2_O) †	93 ± 66.6	86 ± 26.4	0.786
Pmax (cm H_2_O) †	34 ± 30.8	25 ± 23.2	0.220
PQmax (cm H_2_O) †	25 ± 20.1	13 ± 16.6	0.119
Qmax (mL/s) †	13 ± 8.8	15 ± 8.7	0.571
Maximum abdominal voiding pressure (cm H2O) †	42 ± 28.7	55 ± 37.9	0.153
Voiding volume (mL) †	242 ± 124.9	299 ± 134.1	0.115
Postvoid residual (mL) †	28 ± 76.7	12 ± 43.2	0.341
BOOI (cm H_2_O) †	−1 ± 31.8	−12 ± 23.6	0.131
URA (cm H_2_O) †	15 ± 15.6	9 ± 9.8	0.078
BCI (cm H_2_O) †	94 ± 43.7	92 ± 49.2	0.88

* Number (percentage); † mean ± standard deviation; ‡ significant; OAB: overactive bladder; ICIQ: International Consultation on Incontinence Questionnaire; DO: detrusor overactivity; Pmax: maximum voiding detrusor pressure; PQmax: detrusor pressure at Qmax; Qmax: maximum flow rate; BOOI: bladder outlet obstruction index; URA: urethral resistance; BCI: bladder contractility index.

**Table 4:** Relationship between postoperative clinical and urodynamic variables and presence of postoperative detrusor overactivity.

	Postoperative OAB (*n* = 24)	No Postoperative OAB (*n* = 32)	Significance
Number of ATOMS adjustments †	2.1 ± 1.80	0.8 ± 1.10	0.007 ‡
Final cushion volume †	20 ± 4.6	15 ± 4.2	0.001 ‡
Final pad number per day †	0.6 ± 0.92	0.3 ± 0.88	0.359
Final incontinence amount (mL/day) †	49 ± 79.1	32 ± 87.7	0.498
Postoperative complications *	5 (24%)	1 (4%)	0.049 ‡
Continence *	13 (62%)	23 (85%)	0.065
ICIQ score †	7 ± 3.2	3 ± 4.1	0.000 ‡
Cystometric capacity (mL) †	143 ± 82.3	243 ± 95.4	0.000 ‡
Bladder compliance (mL/cm H_2_O) †	71 ± 81.5	151 ± 124.2	0.111
DO *	21 (87%)	19 (59%)	0.02 ‡
Volume at first IC (mL) †	107 ± 70.6	187 ± 93.5	0.004 ‡
Maximum IC pressure (cm H_2_O) †	52 ± 25.2	43 ± 25.4	0.269
Stress urinary incontinence *	20 (83%)	23 (72%)	0.249
Abdominal leak point pressure (cm H_2_O) †	164 ± 46.4	167 ± 73.1	0.899
Pmax (cm H_2_O) †	46 ± 33.1	36 ± 26.5	0.213
PQmax (cm H_2_O) †	30 ± 19.0	24 ± 4.2	0.546
Qmax (mL/s) †	7 ± 4.0	12 ± 9.5	0.003 ‡
Voiding volume (mL) †	106 ± 56.5	202 ± 117.9	0.000 ‡
Postvoid residual (mL) †	24 ± 39.9	24 ± 49.9	0.996
BOOI (cm H_2_O) †	17 ± 23.1	2 ± 32.6	0.055
URA (cm H_2_O) †	23 ± 16.7	13 ± 11.4	0.009 ‡
BCI †	61 ± 21.7	86 ± 49.7	0.0012 ‡

* Number (percentage); † mean ± standard deviation; ‡ significant; ATOMS: adjustable trans-obturator male system; ICIQ: International Consultation on Incontinence Questionnaire; OAB: overactive bladder; DO: detrusor overactivity; IC: involuntary contraction; Pmax: maximum voiding detrusor pressure; PQmax: detrusor pressure at Qmax; Qmax: maximum flow rate; BOOI: bladder outlet obstruction index; URA: urethral resistance; BCI: bladder contractility index.

**Table 5:** Regression model of variables that influence the presence of postoperative overactive bladder.

	Multivariate Coefficient	Statistical Significance	Determination Coefficient of the Model (R2)
Volume at first IC	−0.008	0.003	0.343
Postoperative URA	0.062	0.023	

IC: involuntary detrusor contraction; URA: urethral resistance.

## Data Availability

Full data will be shared by the corresponding author upon reasonable request.
